# 

**Published:** 1856-07

**Authors:** 


### THE JOURNAL OF PUBLIC HEALTH.

### CONTENTS OF No. VI.

State Medicine in Great Britain .................................... 105
Parasites of the Human Body.......................................... 113
Physical Education of Women ........................................ 120
Lord Bacon's Sanitary Philosophy .................................. 126
Diseases of Overfed Convicts ........................................ 137

**EPITOME OF SANITARY LITERATURE:**

Defective Ventilation of Hospitals .................................. 132
Cholera in Oxford ........................................................ 134
Medical Selection of Lives for Assurance .......................... 135
Kaffir Doctors.............................................................. 136

**ORIGINAL COMMUNICATIONS:**

Nursery Government in its Sanitary Aspects. By T. H. Barker, M.D. (*Continued from p.* 58) .................................... 143

Fermenting Alvine Excretions as a source of Disease. By C. H. F. Routh, M.D. 153

**SANITARY AND SOCIAL SCIENCE:**

*Reports on Cities, Towns, and Districts.*—Hastings.—Canterbury ........ 165

**PROGRESS OF EPIDEMICS in Twenty-Seven Towns and Districts** .......... 170

**SANITARY LEGISLATION:**

Notices of Bills and Returns, and of the Report of Mr. Simon on Cholera .. 191

**TRANSACTIONS of the EPIDEMIOLOGICAL SOCIETY of London:**

The Geographical Distribution of Health and Disease, in connexion chiefly with Natural Phenomena. By A. Keith Johnston, Esq., F.R.S.E... 25
Proceedings of the Society ............................................... 64

